# Nutrigenomics in Dairy Cattle: Causes and Implications

**DOI:** 10.1155/vmi/3660248

**Published:** 2026-11-02

**Authors:** Negasa Tamasgen, Bacha Asefa

**Affiliations:** ^1^ Department of Animal Sciences Wollega University P.O. Box 395 Nekemte Ethiopia wollegauniversity.edu.et; ^2^ Livestock and Fisheries Agricultural Office Choman Guduru Oromia Ethiopia

**Keywords:** dairy cattle, epigenetics, fatty acids, feed intake, gene expression, milk fat, nutrigenomics, nutritional genomics, transcription factors

## Abstract

Nutrigenomics is a rapidly developing interdisciplinary field that investigates the interactions between nutrients and the genome, with particular emphasis on how dietary components regulate gene expression and metabolic pathways. In dairy cattle, nutrigenomics has gained increasing attention due to its potential to improve milk production, metabolic efficiency, health, reproduction, and overall animal performance. Nutrients and bioactive dietary compounds can influence gene activity directly through transcriptional regulation or indirectly through epigenetic mechanisms such as DNA methylation, histone modification, and noncoding RNA‐mediated gene regulation. These nutrigenomic interactions affect important biological processes including lipid metabolism, adipogenesis, immune response, mammary gland development, and milk synthesis. Advances in omics technologies including transcriptomics, proteomics, metabolomics, and next‐generation sequencing have enhanced understanding of the molecular responses of dairy cows to nutritional interventions. Dietary fatty acids, vitamins, methyl donors, and short‐chain fatty acids such as butyrate have been shown to regulate key transcription factors including peroxisome proliferator–activated receptors (*PPAR*s), sterol regulatory element binding proteins (*SREBPs*), and liver X receptors (LXRs), thereby modulating metabolic and physiological functions. Nutritional interventions, feed restriction, and supplementation produce tissue‐specific molecular responses in the liver, adipose tissue, mammary gland, and immune system, demonstrating interaction between diet, physiological state, and metabolic regulation. Despite these advances, challenges with genetic and physiological variability, tissue‐specific responses, integration of multi‐omics data, and translation of molecular findings into practical feeding strategies remain unresolved. Future integration of nutrigenomics with microbiome research, multi‐omics, artificial intelligence, and precision livestock technologies may facilitate nutritional strategies to improve dairy cattle health, productivity, feed efficiency, and product quality.

## 1. Introduction

Nutrigenomics is an interdisciplinary field that investigates how nutrients and other dietary components interact with the genome to regulate gene expression, cellular metabolism, and phenotypic responses. In dairy cattle, nutrigenomics provides a molecular framework for understanding how dietary nutrients influence metabolic processes associated with nutrient utilization, lactation, milk synthesis, animal health, and production efficiency, while genetic variation can contribute to differences in nutritional responses among animals [1, 2]. Nutrients and their metabolites can act as signaling molecules that modify transcriptional and regulatory networks, thereby influencing genes involved in lipid, protein, carbohydrate, and energy metabolism. Recent advances in high‐throughput technologies have further expanded dairy nutrigenomics from individual gene expression studies toward integrated genomics, transcriptomics, epigenomics, metabolomics, proteomics, and single‐cell approaches [3]. These approaches are increasingly being used to elucidate the molecular mechanisms underlying milk production and composition and to identify candidate genes, regulatory elements, and metabolic pathways associated with dairy traits [2, 4].

Nutrigenomics has contributed substantially to understanding the molecular regulation of lipid metabolism and milk fat synthesis in dairy cattle. Mammary lipid synthesis is regulated by complex interactions among dietary fatty acids, ruminal biohydrogenation, systemic lipid metabolism, and mammary gene expression [5]. In particular, diet‐induced milk fat depression (MFD) is associated with altered ruminal biohydrogenation and increased production of specific fatty acid intermediates, which can inhibit mammary de novo fatty acid synthesis and milk fat secretion [6]. In dairy cattle, these nutrient gene interactions influence metabolic processes involved in energy utilization, lipid and protein metabolism, mammary gland function, milk synthesis, and adaptation to changes in nutritional supply [7]. Milk fatty acids originate from both *de novo* synthesis in the mammary gland and uptake of preformed fatty acids from the circulation; short‐ and medium‐chain fatty acids are predominantly synthesized within the mammary gland, whereas most long‐chain fatty acids are derived from dietary lipids or mobilized body reserves [8]. Recent transcriptomic and multi‐omics studies have demonstrated that dietary factors can modify the expression of genes involved in fatty acid uptake, *de novo* lipogenesis, desaturation, and triglyceride synthesis, thereby influencing milk fat synthesis and composition [9].

Importantly, nutrigenomic effects extend beyond individual bioactive compounds, as dietary energy supply and specific nutrients can influence gene expression and metabolic regulation through interactions with transcription factors (TFs), nutrient‐sensing pathways, and other regulatory mechanisms [7]. These interactions provide a broader framework for understanding how nutritional factors influence cellular metabolism, tissue function, and metabolic adaptation in dairy cattle. Nutrients can influence gene expression and tissue function through interactions with TFs, signaling pathways, and epigenetic regulatory mechanisms [10]. This review examines current evidence on how nutritional factors regulate gene expression and molecular pathways in dairy cattle and how these nutrient gene interactions contribute to variation in metabolism, animal performance, milk composition, and product quality.

## 2. Advances in Nutrigenomic Methodologies

Understanding how individual nutrients, dietary combinations, feed additives, and overall diet composition regulate gene expression requires technologies capable of capturing molecular responses across multiple biological levels. RNA sequencing (RNA‐seq) has become an important approach for characterizing genome‐wide transcriptional responses to nutritional interventions, while advances in proteomics, metabolomics, epigenomics, and metagenomics have expanded nutrigenomic research beyond gene expression alone [11].

Multi‐omics approaches integrate several biological levels, including genes and gene expression (transcriptomics), proteins and their abundance and functions (proteomics), and metabolites and the biochemical pathways in which they participate (metabolomics). In livestock research, the integration of these approaches is increasingly being used to understand complex traits related to production, health, reproduction, feed efficiency, and responses to nutrition. In ruminants, these technologies have also provided new insights into how dietary components influence metabolism, animal performance, and the nutritional and functional quality of animal products [12]. In dairy cattle, multi‐omics approaches are particularly useful because nutritional responses are not confined to a single tissue or biological pathway. Changes in nutrient supply can simultaneously affect gene expression, protein activity, and metabolite concentrations in tissues such as the mammary gland, liver, and adipose tissue. Examining these molecular responses together can therefore provide a clearer understanding of how dietary nutrients influence milk synthesis, energy metabolism, metabolic health, feed efficiency, and milk composition [13, 14]. More recently, dairy research has begun to combine multi‐omics data with rumen microbiome information and advanced computational approaches, providing a broader view of the interactions among diet, rumen microorganisms, host metabolism, and animal performance [14].

In brief, transcriptomics enables the analysis of changes in mRNA expression profiles, proteomics examines variations in protein abundance and function, and metabolomics evaluates shifts in metabolite concentrations and metabolic pathways. Beyond their application in nutrigenomics, these approaches are increasingly integrated to investigate complex phenotypes in livestock, including feed efficiency, body composition, metabolic adaptation, and nutritional responses [15].

The activation of TFs can be investigated using gene reporter assays, in which a promoter or regulatory DNA region containing specific TF response elements is linked to a reporter gene. Following introduction of the construct into cells, TF activity can be assessed by quantifying reporter‐gene expression. Gene reporter assays can be performed using transient or stable transfection systems. Luciferase‐based reporter assays are widely used because they provide sensitive and quantitative measurements of transcriptional activity. Firefly luciferase is commonly used as the experimental reporter, while Renilla luciferase can be coexpressed as an internal control to normalize differences in transfection efficiency and other experimental variation. Dual‐luciferase systems therefore provide a robust approach for evaluating transcriptional regulation and validating the functional activity of candidate regulatory elements [16].

Recent advanced technologies have shifted dairy nutrigenomics from bulk tissue transcriptomic analyses toward cell‐resolved and integrative multi‐omics approaches. Single‐cell RNA sequencing (scRNA‐seq) has enabled characterization of cellular heterogeneity within bovine mammary and immune tissues that is difficult to resolve using conventional bulk RNA‐seq. Reference [17] applied scRNA‐seq to bovine milk somatic cells and primary mammary epithelial cells, demonstrating distinct epithelial and immune cell populations and providing a framework for investigating mammary function at cellular resolution. Subsequent studies have applied single‐cell transcriptomics to bovine peripheral blood mononuclear cells, monocytes, and lymph node immune populations, revealing previously unrecognized heterogeneity and cell‐specific responses to inflammatory stimulation [18, 19]. More recent studies have further expanded the single‐cell landscape of bovine milk somatic cells and immune tissues, demonstrating the increasing potential of this approach for understanding lactation, mammary immunity, and disease resilience. These developments complement conventional nutrigenomic approaches by enabling nutritional and physiological responses to be attributed to specific cell populations rather than to heterogeneous tissues.

Current evidence demonstrates that the bovine host genome can influence the composition and functional potential of the rumen microbiome, including microbial genes associated with methane production [20]. In dairy cattle, integrated host genome and microbiome analyses have subsequently been applied to feed efficiency, demonstrating that genome microbiome interactions contribute to variation in dry matter intake, milk energy, and residual feed intake. At the same time, artificial intelligence and machine learning methods are increasingly being used to integrate complex biological and phenotypic datasets. For example, Reference [21] applied feature engineering and ensemble machine learning methods to characterize the contribution of the rumen microbiome to feed and milk production efficiency in dairy cows. Machine learning approaches have also been used to predict metabolic status from milk infrared spectra, including blood metabolites related to energy metabolism, oxidative stress, and inflammation [22]. More broadly, recent dairy science research indicates that integration of genomics, metagenomics, metabolomics, proteomics, and high‐throughput phenotyping with machine learning may improve prediction of milk composition, metabolic status, and production efficiency. Thus, the developing direction of dairy nutrigenomics is moving beyond single omics association studies toward cell‐resolved, microbiome‐aware, and AI‐assisted multi‐omics frameworks for precision nutrition and management.

## 3. Nutriepigenomics and Epigenetics Mechanism in Dairy Cows

Nutriepigenomics examines how nutrients and bioactive dietary compounds influence gene regulation and phenotype through epigenetic mechanisms, including DNA methylation, histone modifications, and noncoding RNAs. Unlike the broader field of epigenomics, which investigates genome‐wide epigenetic patterns and their regulation, nutriepigenomics specifically focuses on the effects of nutritional exposure on these regulatory processes. Recent evidence indicates that dietary factors such as methionine, lysine, choline, folate, and other bioactive compounds can modify epigenetic regulation in dairy cattle, potentially affecting genes and pathways involved in metabolism, mammary gland function, milk production and composition, immune function, and reproduction [10]. Nutritional influences on the epigenome are particularly important during maternal, fetal, and early‐life development, when epigenetic programming may contribute to persistent changes in gene expression and subsequent phenotypic outcomes. Recent reviews further indicate that epigenetic mechanisms can connect environmental and nutritional conditions with economically important traits such as growth, metabolism, disease resistance, reproduction, and milk production in livestock [23]. Thus, variation in nutritional exposure may contribute to differences in epigenomic profiles and phenotypic responses even among animals with similar genetic backgrounds, highlighting nutriepigenomics as an important mechanism linking diet, gene regulation, and dairy cattle performance.

Despite increasing interest in nutritional epigenetics, the mechanisms through which specific nutrients regulate DNA methylation, histone modifications, and noncoding RNA expression in dairy cattle remain incompletely understood [24]. Maternal nutrition during gestation is of particular interest because nutrient availability can influence fetal development and may induce persistent molecular changes that affect offspring metabolism, immune function, growth, and subsequent productive performance. Recent studies in cattle have demonstrated that maternal nutritional status can modify DNA methylation and microRNA profiles in fetal tissues, providing evidence that maternal diet can influence developmental programming through epigenetic mechanisms [25]. In addition, recent research on maternal nutritional management during pregnancy has highlighted effects on offspring metabolic and immune‐related pathways, although the extent to which these epigenetic changes translate into long‐term production and health outcomes remains to be fully established [26].

Epigenetic mechanisms constitute an additional layer of gene regulation that controls transcriptional activity without altering the underlying DNA sequence. These mechanisms are highly dynamic and tissue‐specific and play important roles in cellular differentiation, development, and metabolic adaptation [10]. In livestock, epigenetic regulation is increasingly recognized as an important mechanism through which environmental and nutritional factors can influence gene expression and phenotype. The major epigenetic mechanisms include DNA methylation, histone modifications, chromatin remodeling, and regulation by noncoding RNAs (ncRNAs) [27]. During embryonic and postnatal development, dynamic changes in these mechanisms contribute to cell‐type‐specific gene expression and tissue differentiation. In dairy cattle, recent studies have demonstrated that nutritional interventions can modify DNA methylation, miRNA expression, and gene‐regulatory pathways in mammary and fetal tissues, providing evidence that epigenetic mechanisms may mediate nutritional effects on development, metabolism, and productive traits [25].

Furthermore, the nutrient–epigenome–phenotype relationships are complex and difficult to establish due to variations in tissue type, developmental stage, nutritional dose, genetic background, and experimental design. In conclusion, the evidence currently available suggests that nutritional epigenetic regulation plays an important role, but more integration of epigenomic data with transcriptomics, metabolomics, microbiome profile, and longitudinal measurements of phenotypes is necessary to identify which epigenetic responses are transient and which have functional consequences for dairy cattle health, productivity, and product quality.

### 3.1. Deoxyribonucleic Acid (DNA) Methylation

DNA methylation is one of the most extensively studied epigenetic mechanisms. It primarily involves the addition of a methyl group to cytosine residues, particularly at cytosine–guanine dinucleotides (*CpG* sites). Genomic regions with a high density of *CpG* sites are known as *CpG* islands, many of which occur in or near gene promoter regions. In general, increased DNA methylation at promoter‐associated *CpG* islands is associated with reduced transcription, whereas promoter hypomethylation is often associated with greater gene activity. However, the functional consequences of DNA methylation depend on its genomic location, developmental context, and interaction with other regulatory mechanisms [28].

Maternal nutritional status, including the availability of methyl donors, can influence epigenetic programming during fetal development and may consequently affect offspring metabolic and physiological outcomes. In beef cattle, variation in maternal body weight gain during early gestation was associated with changes in DNA methylation and miRNA expression in fetal liver. The differentially methylated genes were mainly related to anatomical development and transcriptional regulation, whereas miRNA‐associated targets were enriched in processes involving embryonic development, energy metabolism, and mineral transport [25]. Similarly, maternal undernutrition at 60% of nutritional requirements, compared with a higher nutritional supply of 120% of requirements, induced widespread alterations in fetal thymus DNA methylation detected using whole genome bisulfite sequencing (WGBS). These epigenetic changes were accompanied by differences in gene expression, amino acid metabolism, and immune‐related pathways [29].

In dairy cattle, DNA methylation and other epigenetic mechanisms contribute to the regulation of gene expression in response to physiological, environmental, and nutritional conditions. Recent evidence demonstrates that DNA methylation patterns are associated with mammary gland function, milk production, and udder health, while genome‐wide analyses have also identified differential methylation regions associated with lactation status and milk traits [30]. In addition, microRNAs (miRNAs) are important post‐transcriptional regulators that influence mRNA stability and translation and participate in the regulation of mammary development and milk fat and milk protein synthesis. Recent multi‐omics analysis of cattle identified numerous miRNAs that were differentially expressed between lactating and nonlactating states, together with regulatory relationships between miRNAs and their target genes, highlighting their potential importance in controlling milk production traits [4].

Variability between studies may be due to differences in tissue type, developmental stage, nutritional treatment, genetic background, and genomic location. Therefore, combining DNA methylation and miRNA profiles with transcriptomics, metabolomics, and phenotypic data will play a crucial role in identifying nutritionally relevant DNA methylation and miRNA changes and elucidating their potential use in precision nutrition and dairy cattle production.

### 3.2. Histone Modification

Histone proteins package DNA into chromatin, and their post‐translational modifications (PTMs) influence chromatin structure, DNA accessibility, and transcriptional activity. Common histone modifications include acetylation, methylation, phosphorylation, and ubiquitination, which can promote or repress gene transcription depending on the specific modification and genomic context [31]. Histone acetylation generally promotes gene transcription by relaxing chromatin structure, whereas deacetylation suppresses transcription. In dairy cattle, histone modifications regulate genes involved in lactation, lipid metabolism, immune function, and embryonic development. Nutritional compounds such as short chain fatty acids (SCFAs), especially butyrate, can act as histone deacetylase inhibitors and modify gene expression associated with milk production and metabolism. Histone PTMs are covalent chemical alterations to histone proteins that function as critical epigenetic regulators of chromatin structure and gene expression [32]. Furthermore, histone PTMs are involved in early mammalian development and the regulation of numerous biological functions, while abnormal modification patterns have been associated with the development of various genetic and epigenetic disorders [33].

Histone acetylation is generally associated with transcriptional activation by loosening chromatin structure and enhancing gene accessibility to TFs. In contrast, histone methylation may either activate or repress gene expression depending on the specific amino acid residue modified and the degree of methylation [34]. Nutritional factors can also influence histone modifications and thereby affect lactation performance and milk composition. Several dietary bioactive compounds, including polyphenols and omega‐3 fatty acids, have been reported to modify histone acetylation and methylation patterns in mammary tissues. These epigenetic alterations can regulate the expression of genes involved in milk synthesis, fatty acid metabolism, and mammary gland development. Studies in dairy goats have demonstrated that nutritional manipulation with different dietary fat sources can alter mammary gland expression of lipogenic genes and modify milk fatty acid composition, providing evidence that dietary factors can regulate molecular pathways involved in milk fat synthesis and quality [35].

Overall, histone modifications represent a reasonable molecular mechanism through which nutritional signals can influence gene regulation and cellular function in dairy cattle. Future studies integrating histone profiling and chromatin‐accessibility assays with transcriptomics, metabolomics, and nutritional and phenotypic measurements will therefore be important for determining how specific dietary components influence histone‐mediated regulation and how these mechanisms contribute to dairy cattle health, lactation, metabolism, and product quality.

### 3.3. Noncoding RNA (ncRNA)‐Associated Gene Regulation

Noncoding RNAs (ncRNAs), including microRNAs (miRNAs) and long noncoding RNAs (lncRNAs), are important regulators of gene expression at transcriptional and post‐transcriptional levels. In dairy cattle, ncRNAs participate in mammary gland development and function and contribute to the regulation of milk synthesis, lipid metabolism, immune responses, and mammary health. Recent transcriptomic and multi‐omics studies have identified extensive networks of miRNAs and lncRNAs associated with lactation and milk production traits, while differential expression of ncRNAs has also been observed in cows with mastitis, suggesting important roles in inflammatory and immune responses of the mammary gland [36, 37]. These findings highlight the potential of integrating computational prediction with functional validation to elucidate ncRNA‐mediated regulatory networks underlying mammary gland physiology, disease resistance, and milk composition.

## 4. Gene Expression and Regulation

TFs regulate gene expression by binding to specific regulatory sequences and either activating or repressing transcription of their target genes. Nutritional and metabolic signals can modify the activity of TFs and their interactions with promoters, enhancers, silencers, and other cis‐regulatory elements, thereby altering the expression of genes involved in metabolism, mammary gland function, milk synthesis, and animal adaptation [38]. Recent advances in transcriptomics and functional genomics have enabled the identification of nutrient‐responsive gene networks and regulatory elements in dairy cattle, providing molecular insights into variation in milk production and composition [39].

Gene expression profiling can therefore be used to evaluate the molecular responses of tissues to nutritional interventions and to identify pathways regulated by nutrient‐sensitive TFs. In dairy cattle, RNA‐sequencing and multi‐omics approaches have identified differentially expressed genes and regulatory networks associated with lactation, milk fat synthesis, milk protein production, and mammary gland development [2, 4]. These approaches provide an important molecular basis for understanding how dietary nutrients influence gene regulation and ultimately affect animal performance and product quality.

## 5. Nuclear Receptors and Dietary Lipid Signaling in Dairy Cows

Intracellular (nuclear) receptors are ligand‐activated TFs that respond to lipid‐soluble signaling molecules, including steroid hormones, thyroid hormones, retinoids, and vitamin D metabolites. Upon ligand binding, these receptors interact with specific DNA response elements to regulate the transcription of target genes. Among these, retinoic acid, an active metabolite of vitamin A, regulates adipocyte differentiation and metabolic function through retinoic acid receptor (RAR) and retinoid X receptor (RXR) signaling. Its effects on adipogenesis are stage‐dependent, with retinoic acid generally inhibiting early adipocyte differentiation while promoting transcriptional programs associated with lipid oxidation and thermogenic function at later stages [40].

Gene expression regulated by nuclear receptors is further modulated by coregulatory proteins. Nuclear corepressors promote chromatin condensation and transcriptional repression, whereas coactivators facilitate transcription by recruiting chromatin‐remodeling and histone‐modifying complexes that increase chromatin accessibility [41]. Retinoic acid signaling has stage‐dependent effects on adipose development; it can promote preadipocyte commitment while inhibiting terminal adipocyte differentiation and lipid accumulation, and it can also promote thermogenic programming in mature adipose tissues [27, 42].

Dietary lipids play a central role in regulating gene expression in metabolically active tissues such as the liver and mammary gland. Beyond serving as energy substrates and structural components, fatty acids act as signaling molecules that modulate TFs involved in lipid metabolism, including peroxisome proliferator–activated receptors (*PPARs*) and sterol regulatory element binding proteins (*SREBPs*) [43]. The *PPAR* family comprises *PPARα, PPARγ,* and *PPARβ/δ*, which regulate diverse aspects of fatty acid oxidation, lipid storage, adipogenesis, and energy metabolism. Similarly, *SREBPs*, particularly SREBP‐1c and SREBP‐2, regulate genes involved in de novo fatty acid synthesis and cholesterol homeostasis. These transcriptional regulators influence the expression of genes involved in long‐chain PUFA biosynthesis, including *FADS1*, *FADS2*, *ELOVL2*, and *ELOVL5*, which encode fatty acid desaturases and elongases, respectively [16]. Polyunsaturated fatty acids (PUFAs) and their metabolites are therefore important nutritional regulators of gene expression, acting through nuclear receptors and other transcriptional pathways to modify lipid metabolism and cellular function [43]. In ruminants, dietary PUFA supplementation can additionally modify mammary gland gene networks associated with lipogenesis, fatty acid desaturation, and milk fat synthesis, demonstrating the importance of lipid‐mediated nutrigenomic regulation in dairy production [44].

SCFAs produced during ruminal fermentation, particularly acetate, propionate, and butyrate, also exert important nutrigenomic effects. These microbial metabolites function not only as energy substrates but also as signaling molecules that influence gene expression, cellular metabolism, immune responses, and epigenetic processes. Butyrate, in particular, can regulate transcription through inhibition of histone deacetylases (HDACs), thereby altering chromatin accessibility and the expression of genes involved in cell proliferation, immune function, and intracellular signaling [45]. In addition, free fatty acid receptors (FFAR2/GPR43 and FFAR3/GPR41) recognize SCFAs and mediate cellular responses through G‐protein‐coupled signaling pathways, including changes in intracellular Ca^2+^, cyclic AMP (cAMP), and mitogen‐activated protein kinase (MAPK) signaling. In ruminants, SCFA receptor signaling represents an important mechanism linking ruminal microbial fermentation with host metabolic and immune regulation [46].

Nutritional interventions using lipid supplements such as fish oil, sunflower oil, linseed oil, and palm oil can differentially influence mammary gland gene expression and milk composition. In dairy cattle, dietary PUFA supplementation has been associated with changes in milk fatty acid composition and the expression of genes involved in fatty acid synthesis, desaturation, elongation, transport, and triglyceride synthesis. However, responses of individual lipogenic genes are not always consistent, indicating that dietary lipids can exert complex, pathway‐specific effects on mammary lipid metabolism [44]. Beyond protein‐coding genes, microRNAs (miRNAs) provide an additional layer of post‐transcriptional regulation by influencing mRNA stability and translation. Dietary and metabolic changes can modify mammary miRNA profiles, potentially affecting pathways involved in lipid metabolism, mammary remodeling, and milk synthesis. In dairy cows, integrated miRNA and mRNA analyses have identified differentially expressed miRNAs and their target genes associated with lipid metabolism and mammary gland function [47]. In particular, feed restriction altered 27 miRNAs and 374 mRNAs in the mammary gland of Holstein cows, with several miRNA mRNA interactions linked to lipid metabolism and mammary remodeling [47]. Such interactions between dietary fatty acids, TFs, protein‐coding genes, and noncoding RNAs highlight the complexity of nutrigenomic regulation in dairy cattle. Therefore, integrating transcriptomic and noncoding RNA data with other omics approaches can provide a more comprehensive understanding of how dietary lipids regulate mammary function, milk composition, and animal metabolic responses [44, 47].

## 6. Nutrigenomic Responses to Plane of Nutrition and Energy Balance

Nutritional management, particularly changes in feed intake, can substantially influence gene expression and metabolic adaptation in dairy cows. Feed restriction alters hepatic and mammary transcriptional programs involved in energy metabolism, lipid mobilization, nutrient utilization, and metabolic adaptation during lactation. For example, nutrigenomic analysis of mid‐lactation dairy cows subjected to feed restriction identified extensive changes in mammary gland gene expression, including 374 differentially expressed mRNAs and 27 differentially expressed miRNAs, with several altered genes and regulatory networks associated with lipid metabolism and mammary tissue remodeling [47]. In addition, the metabolic response to altered nutrient supply involves coordinated changes in hepatic pathways governing gluconeogenesis, fatty acid metabolism, and energy homeostasis, reflecting adaptive mechanisms that help dairy cows cope with periods of reduced nutrient availability [48]. Transcriptomic responses to reduced nutrient availability involve pathways related to lipid metabolism, energy utilization, oxidative processes, and metabolic adaptation, reflecting coordinated mechanisms that help prioritize nutrient use for essential physiological functions and milk production. Alterations in hepatic lipid metabolism are also associated with changes in nuclear receptor signaling, including pathways regulated by PPARs, which coordinate fatty acid oxidation and energy homeostasis [49].

Collectively, current evidence indicates that dietary energy supply and nutritional status exert tissue‐specific nutrigenomic effects involving the liver, mammary gland, adipose tissue, and immune system (Table 1). However, these responses are complex because changes in feed intake are usually accompanied by simultaneous alterations in energy, protein, fatty acid, mineral, and microbial metabolite supply. Consequently, it is difficult to attribute changes in gene expression to a single dietary component. Nutritional regulation therefore likely involves coordinated interactions among TFs, nuclear receptors, signaling pathways, metabolites, and noncoding RNAs [44, 47]. Understanding these interactions is therefore essential for moving nutrigenomics from the description of individual transcriptional responses toward the prediction of how nutritional interventions influence whole‐animal metabolism, milk production, and metabolic health.

**TABLE 1:** Representative in vivo nutrigenomic studies in dairy cattle.

Nutritional intervention	Target tissue	Major DEGs, molecular regulators or pathways	Main phenotypic outcomes	Reference
Sodium butyrate (0, 100, 200 and 300 g/d)	Mammary gland	Increased *PPARγ, SREBF1, ACAC*A, FASN, SCD, FABP3, GPR41, and AKT/mTOR signaling; increased cell‐proliferation genes CCNA2, CCND1, PCNA	Increased milk yield, milk fat yield, fat‐corrected milk and energy‐corrected milk; improved nutrient digestibility	[39]
Rumen‐protected choline + methionine	Liver	Altered hepatic expression of genes involved in one carbon metabolism, choline metabolism and lipid metabolism; responses involved methionine/choline metabolic pathways	Modified plasma and milk choline metabolites and amino acids and hepatic metabolic adaptation during the transition period	[59]
Rumen‐protected choline vs. rumen‐protected nicotinamide	Liver transcriptome	Differential regulation of genes associated with lipid metabolism, oxidative stress, inflammation, and energy metabolism	Demonstrated distinct hepatic molecular responses to choline and nicotinamide during the periparturient period	[60]
Rumen protected choline and methionine	Liver	Changes in genes associated with one carbon metabolism, methionine metabolism, phosphatidylcholine synthesis and lipid metabolism	Evidence for altered hepatic methyl donor metabolism and lipid metabolic adaptation	[59]
Rumen‐protected CLA (LCFA; t10, c12‐CLA)	Liver, mammary gland, and adipose tissue	674 mammary DEGs, 479 liver DEGs, and 614 adipose DEGs; enrichment of PPAR, AMPK, fatty acid metabolism, fatty acid transport, PI3K‐AKT, and insulin signaling	Milk fat percentage decreased from approximately 4.0% to 3.5%; circulating NEFA decreased; molecular changes suggested altered energy partitioning and lipid metabolism	[5]
CLA‐induced milk fat depression	RNA‐Seq of mammary gland, liver and subcutaneous adipose tissue	Mammary DEGs enriched in lipid biosynthesis, fatty acid biosynthesis, and steroid biosynthesis; liver DEGs enriched in PPAR, AMPK, PI3K‐AKT, insulin signaling, and fatty acid metabolism	Reduced milk fat synthesis while modifying hepatic and adipose lipid metabolism; potential implications for energy balance and ketosis prevention	[5]
High concentrate/high starch feeding	Mammary gland	Altered expression of TLR4–NF‐κB, oxidative stress, and lipid‐synthesis pathways; suppression of genes including *ACACA*, *FASN, SCD*, *CD36, FABP3,* and *SREBF1*	Associated with ruminal acidosis, inflammation/oxidative stress, and impaired mammary lipid synthesis	[61]
Periparturient nutritional/metabolic transition	Liver and mammary gland	Transcriptomic regulation involving SREBF2, PPARG, PPARA, mTOR, and insulin/IGF signaling	Molecular adaptation to lactation; altered hepatic lipid metabolism, and mammary remodeling; implications for fatty liver and metabolic adaptation	[61]

### 6.1. Liver

Nutritional status and energy balance exert substantial effects on hepatic gene expression in dairy cows, particularly during the transition from late gestation to early lactation. Recent RNA‐sequencing analysis of liver biopsies from cows identified extensive transcriptomic remodeling between the prepartum and postpartum periods, with 1475 genes upregulated and 1199 genes downregulated after calving. Functional analyses indicated activation of glucose, lipid, and amino acid metabolism, with increased gluconeogenesis and fatty acid oxidation and prominent involvement of PPAR and adipocytokine signaling [50].

Negative energy balance (NEB) is likewise associated with substantial changes in hepatic gene expression networks. A systematic review and meta‐analysis integrating 20 studies identified pathways associated with NEB and subclinical or clinical ketosis and highlighted *PPARA* and *ACACA* as important genes involved in lipid metabolism and fatty acid synthesis [51]. In addition, transcriptomic analyses linking metabolic phenotypes with hepatic gene expression have shown that cows experiencing more severe energy imbalance exhibit altered expression of genes involved in fatty acid uptake and oxidation, including *CD36*, *CPT1*, *KLF11*, and *PDK4*, indicating impaired glucose homeostasis and dysregulated hepatic lipid metabolism [52].

Recent experimental evidence further demonstrates that NEB activates adaptive pathways in the liver. Starvation‐induced NEB increased hepatic expression of *PPARA* and genes involved in fatty acid oxidation and ketogenesis, while lipogenic genes were comparatively unchanged, suggesting a shift toward lipid utilization and ketone‐body production rather than generalized activation of hepatic lipogenesis [53]. Complementary metabolomic and proteomic analyses have similarly demonstrated increased gluconeogenesis, tricarboxylic acid‐cycle activity, amino acid degradation, fatty acid oxidation, AMPK signaling, and PPAR signaling in postpartum cows [54]. Collectively, these findings indicate that the liver responds to changes in nutrient supply and energy balance through coordinated transcriptional and metabolic reprogramming that supports glucose production, fatty acid utilization, and metabolic adaptation during early lactation.

### 6.2. Adipose Tissue

Prepartum nutritional status has a substantial influence on the transcriptional adaptation of adipose tissue during the transition period. More recent evidence has strengthened these observations using transcriptomic approaches demonstrating that prepartum energy overfeeding markedly altered the subcutaneous adipose tissue transcriptome of cows, with the greatest differences occurring before calving [55]. Overfeeding primarily affected pathways related to lipid metabolism, fatty acid synthesis, triglyceride accumulation, adipocyte function, and PPAR signaling, indicating that excess prepartum energy promotes a transcriptional state favoring lipid storage. Interestingly, many of these transcriptional effects were transient and diminished after parturition, suggesting that adipose tissue responds strongly to the immediate prepartum nutritional environment but does not necessarily retain a persistent “nutritional memory” comparable with that described for hepatic adaptations.

The effects of prepartum nutrition are closely associated with body condition and adipose insulin sensitivity. Cows with greater body condition exhibit altered expression of genes involved in lipid metabolism and insulin signaling, and high adiposity is associated with reduced insulin responsiveness and greater postpartum lipid mobilization [56]. It interacted with periparturient adipose gene expression and lipid metabolism, supporting the concept that overconditioned cows enter the transition period with a metabolically distinct adipose phenotype [56]. Similarly, transcriptomic profiling by Salcedo‐Tacuma et al. [57] revealed extensive periparturient remodeling of adipose tissue involving lipid metabolism, inflammation, extracellular‐matrix organization, and oxidative stress responses, indicating that adipose adaptation extends beyond simple regulation of triglyceride storage and mobilization. Adipose transcriptional responses with metabolic disease reported that ketosis was associated with altered transcriptional adaptation of subcutaneous adipose tissue, including reduced postpartum expression of *PCK1* and *PCK2*, genes involved in glyceroneogenesis and fatty acid re‐esterification [49].

### 6.3. Mammary Tissue and Immune Cells

Nutritional status also influences mammary gland gene expression and milk synthesis in dairy cows. Recent nutrigenomic studies demonstrate that alterations in nutrient availability can modify mammary transcriptional and post‐transcriptional networks involved in nutrient transport, lipid metabolism, milk synthesis, and mammary tissue remodeling. Feed restriction in mid‐lactation dairy cows altered the expression of 374 mRNAs and 27 miRNAs in mammary tissue, with differentially expressed genes and miRNA–mRNA interactions enriched in pathways associated with lipid metabolism and mammary remodeling [47]. Similarly, integrated analyses of mammary miRNAs and mRNAs have identified regulatory networks associated with milk fat and milk protein traits, demonstrating that nutritional and metabolic status can influence mammary function through coordinated regulation of coding and noncoding RNAs [58]. Dietary PUFA supplementation can also modify mammary gland gene networks involved in fatty acid synthesis, desaturation, elongation, transport, and triglyceride synthesis, further demonstrating the nutrigenomic sensitivity of the mammary gland to dietary composition [44]. These responses suggest that nutritional status can influence the ability of dairy cows to adapt to the metabolic and inflammatory challenges associated with early lactation [48, 51].

## 7. Main Transcriptional Factors With Nutrigenomic Potential in Dairy Cows

Nutrients regulate gene expression through several classes of TFs, particularly ligand‐activated nuclear receptors, including PPARs, liver X receptors (LXRs), RXRs, and vitamin D receptors (VDRs). These receptors function as nutrient and metabolite sensors and regulate genes involved in lipid metabolism, glucose homeostasis, cholesterol metabolism, inflammation, and cellular energy balance [62]. In dairy cattle, dietary fatty acids and their metabolites can influence nuclear receptor–dependent transcriptional networks in tissues such as the liver, adipose tissue, and mammary gland. In particular, PUFA‐rich dietary strategies can modify mammary expression of genes associated with PPAR signaling, fatty acid synthesis, desaturation, elongation, and triglyceride metabolism [44].

In addition to ligand‐activated nuclear receptors, *SREBPs* represent important nutrient‐responsive transcriptional regulators. SREBP‐1, encoded primarily by *SREBF1*, regulates genes involved in de novo fatty acid synthesis, whereas SREBP‐2 is predominantly involved in cholesterol biosynthesis and homeostasis. Their activity is regulated by cellular nutrient and lipid status, allowing transcriptional control of metabolic pathways to respond to changes in nutrient availability [63]. Recent nutrigenomic studies in dairy cattle further demonstrate that dietary and metabolic changes can alter transcriptional networks involving SREBF1 and PPARγ in the mammary gland, contributing to variation in lipid metabolism and milk fat synthesis [44].

Ligand‐activated nuclear receptors are particularly important because they can recognize endogenous metabolites derived from dietary nutrients, including fatty acids, oxysterols, retinoids, and vitamin D metabolites. Following ligand binding, nuclear receptors undergo conformational changes that facilitate interactions with coactivators or corepressors and enable regulation of target genes through specific DNA response elements. Nuclear receptors can function as homodimers or heterodimers, frequently with RXR, and their transcriptional activity can also be modified by post‐translational mechanisms and cellular signaling pathways [62]. Thus, nuclear receptors provide an important molecular interface through which nutritional and metabolic signals are translated into tissue‐specific changes in gene expression. Ligand binding promotes receptor activation and recruitment of transcriptional coregulators rather than simply causing all receptors to dissociate from heat‐shock‐protein complexes and translocate to the nucleus. This distinction is important because the classical cytoplasmic‐to‐nuclear translocation model applies particularly well to some steroid receptors but is not a universal mechanism for all nuclear receptors. Consequently, the nutrigenomic activity of PPARs, LXRs, and related receptors should be considered in the context of ligand availability, receptor abundance, coregulator recruitment, chromatin state, and intracellular signaling [44, 62].

## 8. Implication of Nutrigenomics for Dairy Production and Health

Nutrigenomics has important implications for improving dairy cattle health, productivity, milk nutritional quality, and environmental sustainability. Conventional dairy nutrition is largely based on population level estimates of nutrient requirements and assumes that animals with similar physiological characteristics will respond similarly to a given diet. However, substantial variation exists among individual cows in feed intake, nutrient utilization, metabolic adaptation, disease susceptibility, milk composition, feed efficiency, and greenhouse gas emissions. This variation is influenced by genetic background, physiological stage, rumen microbial ecosystem, and interactions between dietary nutrients and host metabolic pathways. Nutrigenomics provides a mechanistic framework for understanding these interactions by linking dietary nutrients with gene expression, TFs, metabolic pathways, and phenotypic responses. Consequently, nutrigenomic approaches can contribute to a transition from generalized feeding recommendations toward precision dairy nutrition in which diets are more closely matched to the biological characteristics and nutritional responses of individual animals [64, 65].

### 8.1. Prevention and Mitigation of Metabolic Disorders

One of the most important implications of nutrigenomics is its potential contribution to the prevention and mitigation of metabolic disorders, particularly during the transition from late pregnancy to early lactation. During this period, the rapid increase in nutrient requirements for milk synthesis is often not matched by a corresponding increase in dry matter intake, resulting in NEB. Excessive NEB promotes mobilization of adipose tissue, increases circulating nonesterified fatty acids (NEFA), and increases hepatic uptake and oxidation or re‐esterification of fatty acids. When the capacity of the liver to process these substrates is exceeded, triglycerides accumulate in hepatic tissue and ketone body production increases, predisposing cows to fatty liver and ketosis [52]. These disorders can subsequently impair feed intake, immune function, reproductive performance, milk production, and overall animal welfare [66].

Nutrigenomics provides an opportunity to investigate the molecular mechanisms underlying differences among cows in their ability to adapt to NEB. Nutrient availability and metabolic intermediates regulate TFs and signaling pathways involved in fatty acid transport, *β*‐oxidation, ketogenesis, gluconeogenesis, insulin signaling, oxidative stress, and inflammation. Pathways involving PPARs, *SREBPs*, AMP‐activated protein kinase (AMPK), and mechanistic target of rapamycin (mTOR) are particularly relevant because they integrate nutritional and metabolic signals [52]. Transcriptomic and metabolomic profiling can therefore identify molecular signatures associated with successful or unsuccessful metabolic adaptation. Recent multi‐omics research has demonstrated that transcriptomic and metabolomic profiles can discriminate physiological and metabolic states in dairy cows and identify pathways associated with energy and lipid metabolism [67].

Nutrigenomics may also contribute to the prevention of subacute ruminal acidosis (SARA). SARA is commonly associated with excessive intake of rapidly fermentable carbohydrates, inadequate physically effective fiber, and prolonged reductions in ruminal pH [68]. However, cows consuming apparently similar diets can differ in susceptibility because of differences in feeding behavior, rumination, saliva production, rumen microbial communities, and epithelial adaptation. Integration of host transcriptomics with rumen metagenomics and metabolomics could therefore help identify molecular and microbial characteristics associated with susceptibility or resilience to SARA [69]. Such information could support diets that maintain favorable ruminal fermentation and microbial protein synthesis while minimizing prolonged reductions in ruminal pH.

An important future application is the development of early warning biomarkers for metabolic disease. Transcriptomic, proteomic, and metabolomic profiles may detect alterations in energy metabolism, inflammation, lipid mobilization, and oxidative stress before conventional clinical indicators become abnormal. Consequently, multi‐omics approaches could facilitate early identification of high‐risk cows and enable targeted nutritional intervention [70]. This is particularly relevant because metabolic disorders frequently occur around calving, when timely intervention can have substantial consequences for subsequent milk production, reproduction, and health.

### 8.2. Nutritional Modification of Milk Fat and Protein Profiles and Relevance to Human Health

A second important implication of nutrigenomics is the potential to manipulate milk composition through targeted nutritional strategies. Milk fat and protein synthesis are influenced by dietary nutrient supply, ruminal fermentation, genetic background, physiological stage, and mammary gland metabolism. Nutrients and their metabolites can act as substrates and signaling molecules that regulate genes involved in *de novo* fatty acid synthesis, fatty acid desaturation and elongation, amino acid transport, and milk protein synthesis [45]. Dietary lipid manipulation is particularly relevant to the modification of milk fatty acid profiles. It can alter ruminal biohydrogenation and the availability of fatty acid substrates for mammary tissue while also influencing the expression and activity of enzymes involved in lipid synthesis and desaturation.

Nutrigenomic pathways involving *PPARs, SREBPs*, and stearoyl‐CoA desaturase (SCD) are important regulators of mammary lipid metabolism [45]. This has potential relevance to human nutrition and the development of value‐added dairy products. Nutritional strategies can increase the concentration of selected omega‐3 fatty acids and conjugated linoleic acid while potentially reducing the relative abundance of certain saturated fatty acids [71]. Thus, nutrigenomics may contribute to the production of milk with an improved fatty acid profile. However, the human health implications should be interpreted carefully. A change in an individual milk fatty acid does not necessarily translate directly into a clinical health benefit because the effect of dairy foods depends on the complete food matrix, food processing, quantity consumed, fatty acid interactions, and the overall dietary pattern [72]. Therefore, future research should connect changes in milk composition with bioavailability and human health outcomes rather than assuming that molecular modification automatically produces a health benefit.

Nutrigenomics is similarly important for milk protein synthesis and nitrogen utilization. Milk protein production depends on the supply of metabolizable protein and essential amino acids to the mammary gland. Amino acids do not merely function as substrates for protein synthesis; they also act as signaling molecules that regulate pathways associated with mTOR, insulin signaling, cellular growth, and protein turnover [73]. Therefore, the balance of essential amino acids can influence the efficiency of conversion of dietary protein into milk protein. Precision amino acid nutrition offers an opportunity to avoid unnecessary crude protein supplementation by supplying limiting amino acids according to individual production requirements [74]. This approach has implications for both milk quality and environmental sustainability. Supplying adequate but not excessive dietary protein can maintain milk protein production while reducing nitrogen losses. Integration of milk protein yield, milk urea nitrogen (MUN), feed intake, amino acid supply, and molecular indicators of protein metabolism may allow diets to be formulated more accurately according to individual cows [75].

Integrated transcriptomic and metabolomic analyses in dairy cows have identified coordinated changes in genes and metabolites associated with lipid metabolism, amino acid metabolism, PPAR signaling, and milk component traits across lactation stages [67]. Such findings demonstrate that combining transcriptomics and metabolomics can provide a more complete mechanistic understanding of how nutritional and physiological changes influence milk composition than either approach alone.

### 8.3. Environmental Sustainability and Mitigation of Enteric Methane

Nutrigenomics also has considerable implications for reducing the environmental footprint of dairy production. Enteric methane (CH_4_) is generated primarily by methanogenic archaea during ruminal fermentation and represents both an important greenhouse gas emission and a loss of dietary energy. Improving feed efficiency and modifying ruminal fermentation can therefore contribute simultaneously to productivity and environmental sustainability [76]. Nutrigenomic approaches can contribute by identifying host genes, metabolic pathways, and molecular phenotypes associated with low methane production. Integration of host genomics with rumen microbiome data, transcriptomics, metabolomics, and individual methane measurements may identify animals that naturally produce less methane or use dietary nutrients more efficiently [77]. Genetic studies have demonstrated measurable variation in methane emission traits among dairy cattle, supporting the potential incorporation of methane‐related phenotypes into breeding programs [78].

Nutritional approaches to methane mitigation include improving forage digestibility, optimizing dietary fiber and carbohydrate composition, manipulating rumen fermentation, using appropriate lipid sources, and incorporating methane mitigating feed additives. However, the response to these interventions can differ substantially among individual cows. Variation in methane production is associated with differences in dry matter intake, diet utilization, rumen fermentation patterns, microbial communities, and host genetics [69]. Recent research indicates that the interaction between the host, diet, and rumen microbiome is central to both feed efficiency and methane production [69].

The combination of nutritional and genetic strategies is particularly promising. For example, cows with a genetically favorable low methane phenotype could be selected for breeding, while dietary interventions could be optimized according to individual responses. Such an approach may be more effective than applying a single methane mitigation strategy uniformly across a herd. Recent findings indicated that substantial reductions in dairy methane emissions are likely to require a combination of genetic selection, dietary reformulation, feed additives, improved production efficiency, and manure management strategies rather than reliance on a single intervention [76]. Importantly, methane mitigation should be evaluated not only as a reduction in total CH_4_ production but also as a reduction in CH_4_ emission intensity, expressed per unit of milk or milk protein produced. A strategy that reduces methane but substantially decreases milk production may provide limited environmental benefit when assessed at the production system level. Therefore, nutrigenomic interventions should be evaluated simultaneously for effects on methane production, feed efficiency, milk yield, milk composition, animal health, and economic performance [76].

## 9. Conclusion

Nutrigenomics has advanced the understanding of how nutrients, dietary patterns, and feed additives interact with the genome to influence metabolism, health, production, and product quality in livestock. Integration of transcriptomics, proteomics, metabolomics, epigenomics, and microbiome analyses is increasingly revealing the biological mechanisms underlying nutritional responses and providing a foundation for more precise nutritional management. However, translating these molecular findings into practical feeding recommendations remains challenging because nutritional responses are influenced by animal genotype, physiological stage, environment, microbiota, and interactions among multiple nutrients. Future research should therefore move beyond descriptive omics studies toward integrated, longitudinal, and mechanistic approaches that connect molecular responses with measurable production, health, and environmental outcomes. Greater use of multi‐omics integration, single‐cell technologies, microbiome–host interaction studies, and artificial intelligence or machine learning approaches could help identify reliable biomarkers and predict individual responses to dietary interventions. Particular attention should be given to validating nutrigenomic biomarkers under commercial production conditions and across breeds, environments, and physiological stages. Research should also prioritize nutritional strategies that simultaneously improve animal health and productivity while reducing methane emissions and nitrogen losses, thereby contributing to the translation of nutrigenomic knowledge into evidence‐based precision feeding strategies and a more sustainable livestock production system.

## Author Contributions

Negasa Tamasgen synthesized the idea, wrote, edited, and approved. Bacha Asefa wrote, edited, and approved.

## Funding

No funding was received for this manuscript.

## Conflicts of Interest

The authors declare no conflicts of interest.

## Data Availability

The data that support the findings of this study are available on request from the corresponding author. The data are not publicly available due to privacy or ethical restrictions.
